# Evaluation and prevention of violence in the emergency department in Lebanon

**DOI:** 10.1186/cc13261

**Published:** 2014-03-17

**Authors:** Z Fadel, N Hachem, C El Ramy, G Abi Nader, D Haykal

**Affiliations:** 1 Sainte Famille University, Batroun, Lebanon

## Introduction

At the hospital we are faced with situations of violence, whether verbal or physical, especially in the emergency department (ED) [1]. The objective of this study is to evaluate the phenomenon of violence at hospitals in Lebanon, especially in the ED, and to recommend techniques to prevent it.

## Methods

A questionnaire consisting of 18 questions was sent to the caregivers in the ED of three randomly selected hospitals in Beirut, Lebanon in 2012. A total of 111 people (nurses, aides, doctors, interns, residents, social workers and security guards) responded to the survey questionnaire.

## Results

The majority of the surveyed people are young women (62%) aged between 20 and 40 years (78%) with a nursing degree (74%) and professional experience<5 years (48%). In total, 59% of respondents have experienced violence in the ED during the night (58%) from the patients (31%) or their companions (68%). The caregivers most affected by the violence are nurses (54%) and employees of reception (46%). Violence can be verbal (threats 47%, insults 36%, criticism 18%) or physical (hitting 43%, slapping 40%, stabbing 17%). The dissatisfaction of the patient in his care (42%) and his anxiety (33%) are the most important factors in the generation of violence that may have repercussions on the care workers and their psychological status.

## Conclusion

Violence in the ED may be due to the heavy workload of the caregivers causing a delay in care. Secondly, patients in the ED may feel insufficiently informed and heard by the nursing staff. The priority given to emergencies depending on the severity and not the order of arrival can be misunderstood [2]. Therefore, we recommend the following actions: encourage caregivers to improve their knowledge and training on the management of patients in emergency situations; train emergency caregivers to mediation, nonviolent communication and managing stressful situations; and increase the number of nurses and security guards in the ED and motivate them to ensure a better quality of care and minimize the delay in their care.
